# COVID-19 in underlying COPD Patients

**DOI:** 10.17179/excli2021-3322

**Published:** 2021-02-05

**Authors:** Krishna Sunkara, Venkata Raj Allam, Shakti D. Shukla, Dinesh K. Chellappan, Gaurav Gupta, Ronan MacLoughlin, Kamal Dua

**Affiliations:** 1 Intensive Care Unit, John Hunter Hospital, Newcastle, NSW, Australia; 2 Department of Medical Biochemistry and Microbiology, Uppsala University, Uppsala, Sweden; 3 Priority Research Centre for Healthy Lungs, Hunter Medical Research Institute (HMRI), University of Newcastle, New Lambton Heights, Newcastle, NSW 2305, Australia; 4 Department of Life Sciences, School of Pharmacy, International Medical University (IMU), Kuala Lumpur, Malaysia; 5 School of Pharmacy, Suresh Gyan Vihar University, Jagatpura, Jaipur, India; 6 Aerogen, IDA Business Park, Dangan, H91 HE94, Galway, Ireland; 7 School of Pharmacy & Biomolecular Sciences, Royal College of Surgeons in Ireland, D02 YN77 Dublin, Ireland; 8 School of Pharmacy and Pharmaceutical Sciences, Trinity College, D02 PN40 Dublin, Ireland; 9 Discipline of Pharmacy, Graduate School of Health, University of Technology Sydney, Sydney 2007, Australia

## ⁯

***Dear Editor,***

The devastating social and economic effects which have resulted from the ongoing global coronavirus pandemic have caused a global health crisis affecting tens of millions of people and pushing scores of them into poverty. The disease is caused by the novel severe acute respiratory syndrome (SARS) coronavirus-2 (SARS-CoV-2), which causes viral pneumonia and is known as coronavirus disease 2019 (COVID-19) (Sohrabi et al., 2020[15]). As of 21^st^ December 2020, more than 77 million people were affected with COVID-19 and nearly 1.6 million people have lost their lives (Coronavirus Worldmeter[5]), with mortality rates being higher in older adults and frail individuals (Chinnadurai et al., 2020[4]). In a recent report Ioannidis and colleagues (2020[8]) reported that the mortality rate among patients of < 70 years of age is less compared with patients above 70 years of age. The disease may either be asymptomatic or symptomatic, with signs varying from common cold, flu like symptoms such as cough, fever, and fatigue to severe shortness of breath, pneumonia, and respiratory failure. In addition to severe clinical course, mortality rates are higher in patients with pre-existing conditions such as coronary vascular diseases, hypertension, and diabetes, immunocompromised conditions, and elderly patients (Zhou et al., 2020[16]). Importantly, patients with underlying respiratory diseases such as chronic obstructive pulmonary disease (COPD) are presumed to be more susceptible to COVID-19 and are most likely to suffer from critical clinical complications, requiring intensive care. 

COPD is one of the leading causes of mortality with >3 million deaths occurring annually. It is a progressive, debilitating disease characterized by airflow limitation resulting from chronic inflammation, airway remodeling, and alveolar damage. COPD is a non-reversible disease, primarily managed by oral/inhaled corticosteroids (ICS) that help reduce inflammatory responses, short- or long-acting bronchodilators which relax airway smooth muscles, increasing the airflow and antibiotics to suppress bacterial exacerbations, reducing the risk of complications (Barnes, 2013[3]). However, COPD patients are highly susceptible to viral exacerbations and are most likely to suffer from COVID-19. Therefore, caring of COVID-19 patients with underlying COPD poses a great challenge.

With the global burden of COPD in people aged >45 years being ~10 % (Singh et al., 2019[14]) and amidst the growing pandemic there is an increasing concern of COPD being a risk factor of COVID-19. Interestingly, findings from a systematic review evaluating published data (number of studies=15; total n= 2473 patients) from China has observed the prevalence of COPD in COVID-19 patients to be around a mere 2 %. Besides, it was also reported that the severity and mortality rates were greater in COPD patients who had COVID-19 when compared with COPD patients who were negative for COVID-19 (Alqahtani et al., 2020[1]). However, several other small cohort studies suggest a higher variation in the vulnerability of COPD patients to COVID-19 ranging from a low of 4 %- to as high as 38 % (Leung et al., 2020[9]). Attaway and colleagues (2020[2]), in their findings from a large cohort study of 15,586 symptomatic patients at the Cleveland Clinic COVID-19 registry, showed that 9.2 % of COVID patients had COPD but on adjustment for covariates, COPD was not found to be a risk factor for COVID-19. The low prevalence of COPD in this cohort indicates a superior study design by authors who prevented sampling bias, as they included only COPD patients and excluded patients with other chronic lung conditions (Sin, 2020[13]). Whilst other likely reasons for the reduced susceptibility of COPD patients to coronavirus may be either due to the precautions taken by COPD patients in minimizing contact with COVID-19 patients or due to a potential beneficial effect of steroid use by COPD patients. However, Attaway and co-workers (2020[2]) have also observed that COPD patients have higher hospitalization, ICU admissions and invasive mechanical ventilation rates. These findings confirm the previous results reported by Guan and colleagues (2020[6]) where they observed that, in 575 hospitals in China, COPD subjects were more than 2.5 times at risk of worsened clinical outcomes and death, suggesting an increased risk of severe COVID-19 in COPD patients.

The most likely reasons for the higher risk of severe clinical course of COVID-19 in COPD patients could be due to the increased protein levels of the SARS-CoV-2 receptor, namely angiotensin converting enzyme-2 (ACE-2) in the lower respiratory tract. ACE-2 is associated with a worsened clinical outcome in COVID-19 patients (Leung et al., 2020[10]). SARS-CoV-2 enters the epithelial cells in the nasal mucosa through the activation of their spike proteins by the transmembrane serine protease 2 protein; the activated spike proteins then bind to the ACE-2 protein and enter the host cells. Using the host cellular machinery, the virus produces daughter virions which are released into the extracellular matrix infecting other cells of the lower respiratory tract thereby enhancing the possibility of detrimental outcomes (Leung et al., 2020[9]). Additionally, viral infections in COPD patients are known to cause secondary bacterial infections which further reinforces the higher risk of severe pneumonia in COVID-19 patients. 

One of the important features of severe COVID-19 is the increased levels of pro-inflammatory mediators leading to systemic inflammation and steroids like dexamethasone are known to reduce inflammation. A randomized controlled trial with systemic dexamethasone showed one-third to one-fifth reduction in mortality in COVID patients requiring invasive mechanical ventilation and oxygen supported non-invasive mechanical ventilation respectively (RECOVERY Collaborative Group et al., 2020[11]). Now the key question is, COPD patients who are reliant on steroids would be more susceptible to COVID-19 infection or whether the use of steroids in COPD patients with COVID-19 would augment or alleviate the severity of COVID-19 infection? In a systematic review, Halpin and colleagues (2020[7]) suggested that the effect of ICS could not be associated with either positive or negative consequences in COPD patients with severe COVID-19 infection. In another study involving the UK electronic health records, Schultze and colleagues (2020[12]) observed a slightly higher mortality rate of 0.09 % in COVID-19 patients with COPD who have used ICS compared with those that used long-acting bronchodilators and found little evidence that supports the protective effect of ICS in COPD patients with coronavirus. But, according to the Cleveland clinic study, 18.3 % of people who tested positive for SARS-CoV-2 were less likely to have used corticosteroids compared to 44.8 % who tested negative, suggesting the potential benefit of steroid usage (Attaway et al., 2020[2]). Though there is no concrete evidence of the protective effect of steroids in COPD patients with COVID-19, steroid usage may be considered judiciously after careful examination of potential risk and benefits. 

In summary, COPD patients are presumably at higher risk of COVID-19 infections, however, the exact figures and risk estimates are expected to be clear when more data will be analyzed and reported. Secondly, there seems to be conflicting data on whether the use of corticosteroids in COPD is protective or not, against the development or severity of COVID-19. This, again, will be clear as more robust data gets published in the coming weeks/months. Finally, it needs to be ascertained as to which COPD phenotype (emphysema predominant or bronchitis predominant) or endotype (neutrophil dominant or eosinophil dominant) is more susceptible to either COVID-19 infection or severity. 

## Conflict of interest

The authors declare no conflict of interest.

## References

[1] Alqahtani JS, Oyelade T, Aldhahir AM, Alghamdi SM, Almehmadi M, Alqahtani AS (2020). Prevalence, severity and mortality associated with copd and smoking in patients with covid-19: A rapid systematic review and meta-analysis. PloS One.

[2] Attaway AA, Zein J, Hatipoğlu US (2020). SARS-CoV-2 infection in the COPD population is associated with increased healthcare utilization: An analysis of Cleveland clinic's COVID-19 registry. EClinicalMedicine.

[3] Barnes PJ (2013). Corticosteroid resistance in patients with asthma and chronic obstructive pulmonary disease. J Allergy Cli Immunol.

[4] Chinnadurai R, Ogedengbe O, Agarwal P, Money-Coomes, S, Abdurrahman AZ, Mohammed S (2020). Older age and frailty are the chief predictors of mortality in COVID-19 patients admitted to an acute medical unit in a secondary care setting- a cohort study. BMC Geriatr.

[5] CoronavirusWorldometer www.worldometers.info/coronavirus/.

[6] Guan WJ, Liang WH, Zhao Y, Liang HR, Chen ZS, Li YM (2020). Comorbidity and its impact on 1590 patients with COVID-19 in China: A nationwide analysis. Eur Respir J.

[7] Halpin DMG, Singh D, Hadfield RM (2020). Inhaled corticosteroids and COVID-19: A systematic review and clinical perspective. Eur Respir J.

[8] Ioannidis JPA, Axfors C, Contopoulos-Ioannidis DG (2020). Population-level COVID-19 mortality risk for non-elderly individuals overall and for non-elderly individuals without underlying diseases in pandemic epicenters. Environ Res.

[9] Leung JM, Niikura M, Yang CWT, Sin DD (2020). COVID-19 and COPD. Eur Respir J.

[10] Leung JM, Yang CX, Tam A, Shaipanich T, Hackett T-L, Singhera GK (2020). ACE-2 expression in the small airway epithelia of smokers and COPD patients: implications for COVID-19. Eur Respir J.

[11] RECOVERY Collaborative Group, Horby P, Lim WS, Emberson JR, Mafham M, Bell JL, (2020). Dexamethasone in hospitalized patients with Covid-19 - Preliminary report. N Engl J Med.

[12] Schultze A, Walker AJ, MacKenna B, Morton CE, Bhaskaran K, Brown JP (2020). Risk of COVID-19-related death among patients with chronic obstructive pulmonary disease or asthma prescribed inhaled corticosteroids: An observational cohort study using the OpenSAFELY platform. Lancet Respir Med.

[13] Sin DD (2020). COVID-19 in COPD: A growing concern. EClinicalMedicine.

[14] Singh D, Agusti A, Anzueto A, Barnes PJ, Bourbeau J, Celli BR (2019). Global strategy for the diagnosis, management, and prevention of chronic obstructive lung disease: the GOLD science committee report 2019. Eur Respir J.

[15] Sohrabi C, Alsafi Z, O'Neill N, Khan M, Kerwan A, Al-Jabir A (2020). World Health Organization declares global emergency: A review of the 2019 novel coronavirus (COVID-19). Int J Surg.

[16] Zhou F, Yu T, Du R, Fan G, Liu Y, Liu Z (2020). Clinical course and risk factors for mortality of adult inpatients with COVID-19 in Wuhan, China: A retrospective cohort study. Lancet.

